# Comparison of the genomic background of *MET-*altered carcinomas of the lung: biological differences and analogies

**DOI:** 10.1038/s41379-018-0182-8

**Published:** 2018-11-20

**Authors:** Roberta Castiglione, Christina Alidousty, Barbara Holz, Svenja Wagener, Till Baar, Carina Heydt, Elke Binot, Susann Zupp, Anna Kron, Jürgen Wolf, Sabine Merkelbach-Bruse, Hans Christian Reinhardt, Reinhard Buettner, Anne Maria Schultheis

**Affiliations:** 1 Institute of Pathology, University Hospital of Cologne, Cologne, Germany; 2 Else Kröner Forschungskolleg Clonal Evolution in Cancer, University Hospital Cologne, Cologne, Germany; 3 Institute of Medical Statistics and Computational Biology, University of Cologne, Cologne, Germany; 4 Department I of Internal Medicine, University Hospital of Cologne, Cologne, Germany; 5 Center for Molecular Medicine, University Hospital of Cologne, Cologne, Germany

**Keywords:** Genetics research, Non-small-cell lung cancer, Translational research

## Abstract

Although non-small-cell lung cancer is a leading cause of cancer-related deaths, the molecular characterization and classification of its genetic alterations has drastically changed treatment options and overall survival within the last few decades. In particular, tyrosine kinase inhibitors targeting specific molecular alterations, among other MET, have greatly improved the prognosis of non-small-cell lung cancer patients. Here, we compare the genomic background of a subset of non-small-cell lung cancer cases harboring either a *MET* high-level amplification (*n* = 24) or a *MET* exon 14 skipping mutation (*n* = 26), using next-generatison sequencing, fluorescence in situ hybridization, immunohistochemistry, and Nanostring nCounter^®^ technology. We demonstrate that the *MET*-amplified cohort shows a higher genetic instability, compared with the mutant cohort (*p* < 0.001). Furthermore, *MET* mutations occur at high allele frequency and in the presence of co-occurring *TP53* mutations (*n* = 7), as well as *MDM2* (*n* = 7), *CDK4* (*n* = 6), and *HMGA2* (*n* = 5) co-amplifications. No other potential driver mutation has been detected. Conversely, in the *MET*-amplified group, we identify co-occurring pathogenic *NRAS* and *KRAS* mutations (*n* = 5) and a significantly higher number of *TP53* mutations, compared with the *MET*-mutant cohort (*p* = 0.048). Of note, *MET* amplifications occur more frequently as subclonal events. Interestingly, despite the significantly (*p* = 0.00103) older age at diagnosis of stage IIIb/IV of *MET*-mutant patients (median 77 years), compared with *MET* high-level amplified patients (median 69 years), *MET*-mutant patients with advanced-stage tumors showed a significantly better prognosis at 12 months (*p* = 0.04). In conclusion, the two groups of *MET* genetic alterations differ, both clinically and genetically: our data strongly suggest that *MET* exon 14 skipping mutations represent an early driver mutation. In opposition, *MET* amplifications occur usually in the background of other strong genetic events and therefore *MET* amplifications should be interpreted in the context of each tumor's genetic background, rather than as an isolated driver event, especially when considering MET-specific treatment options.

## Introduction

Lung cancer is the leading cause of cancer deaths in women and men with more than 230.000 expected new cases and an estimated 150.000 cancer-related deaths in the United States in 2018 [1]. However, the genetic characterization of lung cancer and the subsequent development of targeted treatment approaches have fundamentally changed treatment options for patients [2–5]. Since tyrosine kinase inhibitors targeting aberrant products of gene alterations, such as the anaplastic lymphoma receptor tyrosine kinase (*ALK*), epidermal growth factor receptor (*EGFR*), ROS1 proto-oncogene receptor tyrosine kinase (*ROS*), and RET proto-oncogene receptor tyrosine kinase (*RET*) were introduced [6–10], the prognosis of patients suffering from non-small-cell lung cancer has drastically improved [5]. However, drug resistance, frequently caused by tumor heterogeneity, remains a major concern [11–14]. Over the last few years, tumor heterogeneity has become the topic of many studies, using next-generation sequencing [13, 15, 16]. These findings have changed the perception of current tumor biopsy strategies, the characterization of actionable targets, and treatment planning, to better control resistance in these patients [13, 17, 18].

The MET proto-oncogene receptor tyrosine kinase and its ligand, the hepatocyte growth factor were first characterized in the mid-1980s and their involvement in non-small-cell lung cancer tumorigenesis was first described in the 1990s [6, 19–21]. *MET* alterations are described in about 5% of non-small-cell lung cancer cases [22], and it is known that *MET* alterations alone are sufficient to drive carcinogenesis [23]. *MET* alterations include copy-number gains and amplifications, as well as single-nucleotide variants, insertions/deletions (indels) being the so-called *MET* exon 14 skipping mutations [24–26]. At the time of this study, no MET inhibitors were approved by the US Food and Drug Administration, but currently, a series of MET-targeting compounds are being investigated in clinical trials [27, 28]. Patients with advanced-stage non-small-cell lung cancer are included in MET inhibitor clinical trials, when the tumor shows either an exon 14 skipping mutation or a *MET* high-level amplification, mostly defined as at least ten gene copies per cell [28].

As MET inhibitors were developed to treat both, patients with either *MET* exon 14 skipping mutations or *MET* high-level amplifications [28], we aimed to investigate, if the genomic background of these tumors differs more than expected and might define two distinct biological subtypes.

## Materials and methods

### Case collection

The archive of the Institute of Pathology of the University Hospital of Cologne, Germany was retrospectively searched for non-small-cell lung cancer cases showing high-level *MET* amplifications with gene copy ≥ 10, as previously defined [29] or *MET* exon 14 skipping mutations [26, 30, 31]. Patients showing *MET* high-level amplification as a mechanism of resistance to EGFR tyrosine kinase inhibitors were excluded from further analysis.

According to selection criteria, we were able to identify 86 biopsies of lung tumors originating from 86 patients. Of those, 26 tumors harboring *MET* exon 14 skipping mutation and 24 harboring *MET* high-level amplification presented sufficient clinical follow-up and sufficient material for further analyses. For each of the 50 cases, the material was derived from the biopsy at the time of diagnosis of stage IIIB/IV.

Furthermore, 25 resection samples of primary resectable adenocarcinoma of the lung in early stage were collected. This group was used as an independent validation cohort for *MET* high-level amplification using FISH analysis described above.

Histology was reviewed by two experienced pathologists (AMS and RB) according to the current World Health Organization classification criteria [2]. Prior to the study, approval by the local ethics committee was granted and patients signed written informed consent. All samples were anonymized for further analyses.

### Samples and immunohistochemistry

All samples were fixed in 4% neutral-buffered formalin at room temperature and embedded in formalin (formalin-fixed paraffin-embedded) by routine processing methods. For cases with sufficient material (Table 1), 3 µm-thick tissue sections were cut and stained using standard protocols described in Supplementary Table S1.

**Table 1:** Comparison of lung cancers harboring either *MET* exon 14 mutations or high-level amplification

	General	*MET-*mutant	*MET* high-level amplified	*p*
*Sex*				0.00163*
M	31	12	19	
F	19	14	5	
*Age at diagnosis*				0.00052*
Median	71.5	77	69	
Average	71.2	76	66	
*Range*				
< 60	7	1	6	
60–70	10	4	6	
>70	19	14	5	
*Survival time*				0.0030*
Median	185	366	148	
Average	360	456	251	
*Smoker*				0.00021*
Yes	29	8	21	
No	15	13	2	
n.a.	6	5	1	
*MET score*				0.00077*
0	6	6	0	
1	4	4	0	
2	9	3	6	
3	25	9	16	
n.a.	6	4	2	
*PDL-1 score*				0.50973
Average				
0	11	6	5	
1	3	2	1	
2	4	1	3	
3	6	4	2	
4	1	0	1	
5	14	10	4	
n.a.	12	4	8	
MET *FISH*				0.00001*
Average GCN	8.00	2.73	12.75	
Average ratio MET/CEN7	3.73	1.18	6.18	
MDM2 *FISH*				
Not amplified	43	19	24	
Amplified	7	7	0	
CDK4 *FISH*				
Not amplified	44	20	24	
Amplified	6	6	0	
MYC *FISH*				0.02175*
Not amplified	33	21	12	
Amplified	17	5	12	
TP53 *NGS*				0.00485*
wt	27	19	8	
Mutated	23	7	16	

*FISH* fluorescent in situ hybridization, *GCN* gene copy number, *CEN7* centromere of chromosome 7, *NGS* next-generation sequencing

Bold value significant at p < 0.5

For the assessment of the ALK status, ALK expression was classified as positive if strong granular cytoplasmic staining in tumor cells was present [32]. Positive cases were confirmed using fluorescence in situ hybridization (FISH) following a standardized protocol described below (Fluorescence in situ hybridization).

PD-L1 was graded according to the internal guidelines as described in the literature [33, 34]. Score 0 was given if less than 1% of tumor cells were positive, score 1 if between 1 and 4%, score 2 if between 5 and 9%, score 3 if between 10 and 24%, score 4 if between 25 and 49%, and score 5 if at least 50% of tumor cells expressed PD-L1.

**Fig. 1:**
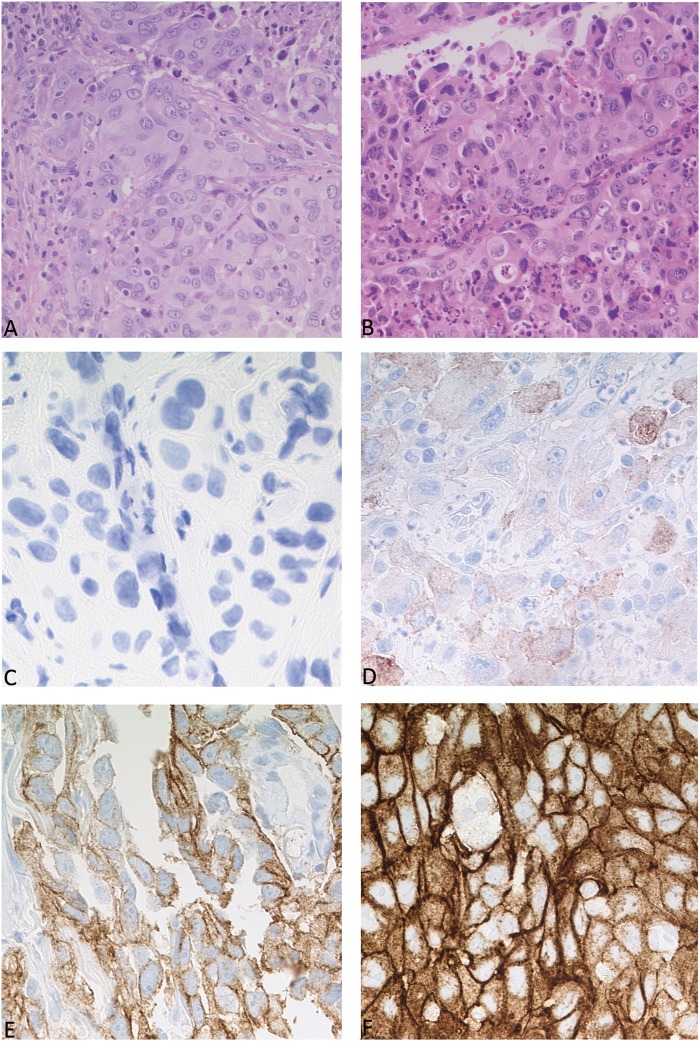
Histology and immunohistochemical analyses. **a** Adenocarcinoma of the lung. **b** Large cell carcinoma of the lung. **c-f** MET immunohistochemistry, score 0 + (**c**), score 1 + (**d**), score 2 + (**e**) score 3+. (**f**) Pictures taken at 20x magnification power

MET expression was determined using immunohistochemistry according to the literature [28, 35, 36] and stained using the clone SP44 (Ventana, Oro Valley, USA), as described in Supplementary Table S1. MET was scored from 0 to 3. Score 3 was assigned if ≥ 50% of tumor cells were stained with strong intensity; score 2 by  ≥ 50% of tumor cells with moderate or higher staining but < 50% with strong intensity; score 1 by ≥ 50% of tumor cells with weak staining but < 50% with moderate or higher intensity; 0 was defined if no staining or < 50% of tumor cells with any intensity. Score 2 and 3 was defined as positive, score 0 or 1 as negative (Fig. 1).

### DNA extraction

For each case, the tumor area was marked on a haematoxylin-eosin stained slide by two senior pathologists (RB, AMS). Six 10-µm-thick sections were cut for each case. After deparaffinization, tumor areas were macrodissected from unstained slides. Tissue was lysed with proteinase K overnight. DNA was purified with the Maxwell^®^ 16 FFPE Plus Tissue LEV DNA Purification Kit (Promega, Mannheim, Germany) on the Maxwell^®^ 16 (Promega), and DNA was eluted in Tris-HCl (pH 7.6) for parallel sequencing approach.

For the NanoString nCounter^®^ analysis, DNA was purified manually using the QIAamp^®^ DNA Mini Kit (Qiagen). Samples were diluted to a working concentration of 150 ng/μl. All extraction procedures were done following the manufacturers’ instructions.

### Parallel sequencing analysis

The DNA content was measured using a quantitative real-time PCR. For multiplex PCR-based target enrichment, the isolated DNA (10 ng each) was amplified with a customized GeneRead DNAseq Targeted Panel V2 (Qiagen, Hilden, Germany), targeting 17 cancer genes most frequently mutated in lung cancer, and the GeneRead DNAseq Panel PCR Kit V2 (Qiagen) according to the GeneRead DNASeq Gene Panel Handbook (Qiagen). The panel comprised a subset of cancer relevant genes including: *ARAF* exons 7, 10, 15, *BRAF* exons 11, 15, *CTNNB1* exon 3, *DDR2* exons 4–19, *EGFR* exons 18–21, *ERBB2* exons 19, 20, *FGFR2* exons 8–10, 12, 17, 20, *FGFR3* exons 7, 10, 15, *KEAP1* exons 2–6, *KRAS* 2–4, *MAP2K1* exon 2, *MET* exons 14, 16–19, *NFE2L2* exon 2, *NRAS* exons 2–4; *PIK3CA* exons 9, 20, *PTEN* exons 1–8, *TP53* exons 5–8.

Libraries were constructed using the Gene Read DNA Library I Core Kit and Gene Read DNA I Amp Kit (Qiagen). After end-repair and adenylation, NEXTflex DNA Barcodes were ligated (Bio Scientific, Austin, TX, USA). Barcoded libraries were amplified and then the final library product was quantified with Qubit dsDNA HS Assay Kit (Thermo Fisher Scientific, Waltham, MA, USA) on the Qubit 2.0 Fluorometer (Thermo Fisher Scientific), diluted and pooled in equal amounts. Finally, 12 pM of the constructed libraries were sequenced on the MiSeq (Illumina, San Diego, CA, USA) with a MiSeq reagent kit V2 (300 cycles) (Illumina) following the manufacturer’s recommendations.

Data were exported as FASTQ files. Alignment and annotation was done using a modified version of a previously described method [37]. BAM files were visualized in the Integrative Genomics Viewer (http://www.broadinstitute.org/igv/, Cambridge, USA). A 5% cutoff for variant calls was used, and results were only interpreted if the coverage was > 200x.

### Fluorescence in situ hybridization

FISH was performed using commercially available FISH probes provided by Zytovision (ZytoVision GmbH, Bremerhaven, Germany) according to manufacturer’s instructions. Further information is available in Supplementary Table S2.

FISH for *MET*, *ROS1*, and *RET* were performed at time of diagnosis. *ALK* FISH was performed in case of any positive or ambiguous immunohistochemistry result. FISH for *MDM2*, *CDK4*, and *MYC* was performed only in selected cases in order to validate gene amplifications as described in the Results section.

Slides were reviewed at high magnification power ( ×63) and scored according to appropriate respective guidelines [29, 38].

### NanoString nCounter assay

All samples were analyzed for copy-number alteration analysis using the NanoString nCounter^®^ platform (NanoString Technologies, Seattle, WA, USA). For detection of copy-number alterations, nCounter Copy Number Variation CodeSets were used with 600 ng of genomic DNA extracted as described above. DNA purity was measured by NanoDropTM 2000c Spectrophotometer (Thermo Fisher Scientific, Waltham, MA, USA), and only DNA samples with an OD A260/280 ratio between 1.7 and 1.9 (indicating optimal purity of DNA) were used for further studies.

DNA was fragmented into small pieces (~500 bp) via AluI digestion (37 °C for 1 h) and subsequently denatured to produce single strands (95 °C for 5 min). Fragmented DNA was hybridized with the CodeSet of 87 genes in the nCounter v2 Cancer CN Assay Kit (NanoString Technologies, Seattle, WA, USA) for 16–18 h at 65 °C and processed according to the manufacturer’s instructions.

The nCounter Digital Analyzer were counted and tabulated the signals of reporter probes. The data were normalized to the invariant control probes and to positive and negative controls in each hybridization reaction. Positive and negative controls as well as the probes were derived from formalin-fixed paraffin-embebbed material, in order to detect any fixation artefacts. Finally, data analysis was performed using nSolver™ Analysis Software 3.0. Copy number was determined by averaging over three probes per region.

Each assay contained 6 positive dsDNA control probes, 8 negative control probes, and 54 invariant genomic control probes designed for autosomal genomic regions predicted not to contain common copy-number alterations.

Based on the manufacturer’s protocol, the gene was considered to be a single copy if the average copy number was below 1.4, two copies if between 1.5 and 2.4, three copies if between 2.5 and 3.4 and continued.

### Statistical analyses

Graphpad Prism software (version 7, Graphpad Software Inc., CA, USA) was used for statistical analyses. To test if the two groups differed in terms of age at diagnosis, smoking status and gender a Student's *t* test was performed. To investigate if the two groups differed in their chromosomal stability, a Brown–Forsythe test was applied as previously described [39]. This inferential method tests two groups for equality in variance. The Brown–Forsythe test is a modification of Levene’s test, but instead of using the mean-based variance, it employs the median-based variance. This makes the Brown–Forsythe test more robust to data skewness and non-normality [40]. For overall survival and one-year survival analyses a log-rank (Mantel–Cox) test was performed.

## Results

### Patient characteristics and histologic classification of tumors

**Fig. 2:**
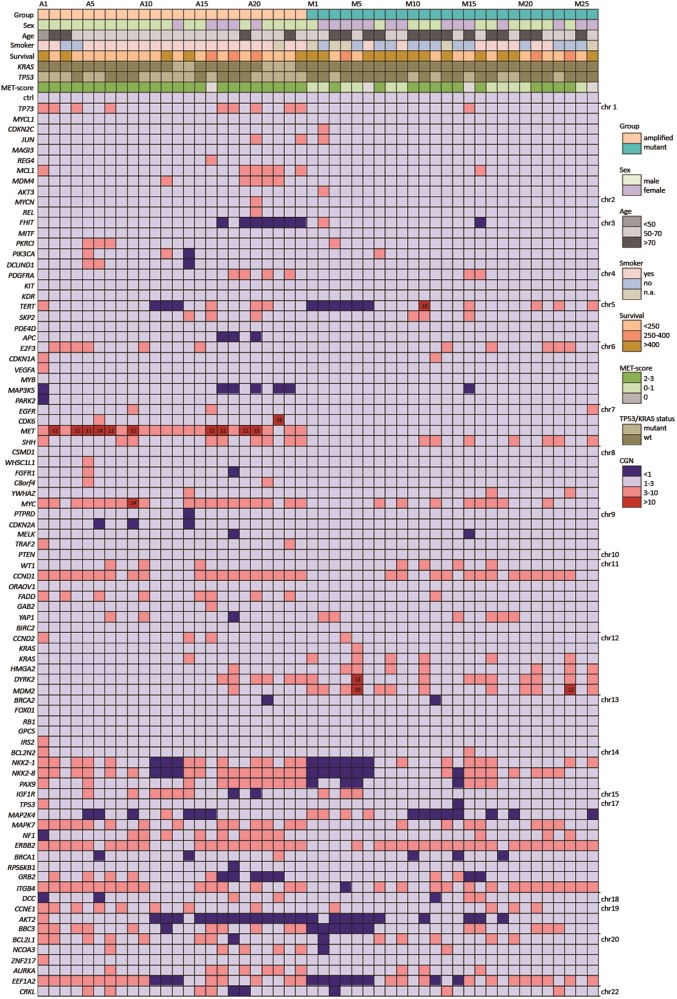
Clinic and genetic features of patients. Overview of the cohort of *MET*-altered lung cancer. Heatmap illustrating the clinical and immunohistochemical features, sequencing results of *KRAS* and *TP53*, copy number variation analysis. Cases are represented in columns; parameters are depicted in rows and color-coded according to the key. ctrl gene copy number control, n.a. not available, wt wild type

Patient characteristics and pertaining statistical analyses are described in detail in Fig. 2, Table 1, and Supplementary Table S3. According to the World Health Organization classification of lung tumors [2], 24 of the 26 *MET-*mutant cases were classified as adenocarcinomas and 2 as squamous cell carcinomas of the lung. Twenty-one of 24 *MET*-amplified cases were considered as adenocarcinomas, two as squamous cell carcinomas, and one as large cell carcinoma of the lung (Fig. 1). Forty-four out of 50 patients were diagnosed at stage IIIb/IV at initial diagnosis and for this reason did not undergo surgery [41]. Due to the limited amount of material provided by a biopsy and the lack of a resection specimen, a further subtyping of the adenocarcinomas was not possible in the routine setting. The kind of biopsy performed is described in Supplementary Table S3.

The median age at diagnosis in the *MET*-mutant cohort was 77 years, significantly higher (*p* = 0.016) than in the high-level amplified cohort (median age 69 years). The *MET* high-level amplified cohort comprised mostly men (81%, *n* = 19), the *MET*-mutant cohort predominantly women (53%, *n* = 14), in line with the current literature [26]. Similar to previous reports [26], we observed a significant predominance of never-smokers (*p* = 0.0002) in the *MET-*mutant cohort. Conversely, the *MET-*amplified group was dominated by smokers (Table 1).

Due to the high heterogeneity of treatment received, any further stratification of patients by therapy was not possible. However, according to ANOVA test, the two groups did not differ significantly in treatment at *p* < 0.05. No patients included in the study received MET-targeted therapy.

### Immunohistochemistry

ALK immunohistochemistry was used as a screening method for the detection of any potential *ALK* translocation, according to the literature [42]. A validated protocol established for the clinical routine setting was applied. Any positive or equivocal immunohistochemistry results were further analyzed, using FISH. In none of the cases, an *ALK* translocation was identified.

MET status was evaluated immunohistochemically by two board-certified pathologists, blinded to genomic or clinical data (Fig. 1). The entire *MET*-amplified cohort showed high consistency with the FISH results: all amplified cases were scored as score 2 (25%, *n* = 6) or 3 (67%, *n* = 16). For two cases (8%) immunohistochemical analysis was not possible, due to the lack of available material. In contrast, in the *MET*-mutant cohort MET-immunohistochemistry showed heterogeneous results. In six of 26 cases (23%) MET was scored as 0, in four (15%) as score 1, in three (12%) as score 2, in nine (35%) as score 3. In four cases (15%), immunohistochemistry was not performed, due to lack of sufficient material.

PD-L1 status showed highly variable results in both cohorts, indicating no detectable relationship between *MET* aberration and PDL-1 expression. In the *MET*-mutant cohort, six of 26 cases (23%) were scored for PD-L1 as 0, 2 (8%) as score 1, one (4%) as score 2, four (15%) as score 3, none as 4 and ten cases (39%) as score 5. In the *MET*-amplified cohort, five of 24 cases (21%) were scored as 0, one (8%) as score 1, three (13%) as score 2, two (8%) as score 3, one (4%) as score 4, and four (17%) as score 5 (Table 1 and Supplementary Table S3). No significant difference was detected among and between the cohorts (*p* = 0.510).

### Targeted next-generation sequencing

All *MET* exon 14 skipping mutations were localized between nucleotide 2842 and 3082. As described in Table 1, no co-occurring *MET* mutation was detected in the *MET*-amplified cohort. The *MET-*mutant cohort contained seven (26.9%) cases with concurrent *TP53* mutation leading to a non-functional protein. No concurrent driver mutations were detected in this cohort.

*TP53* mutations occurred with higher frequency in the *MET*-amplified (67%, *n* = 16), compared with the *MET*-mutant cohort (27%, *n* = 7, *p* = 0.0048). Only the former cohort was characterized by a high frequency of other co-occurring pathogenic driver mutations, such as *KRAS* (17%, *n* = 4) and *NRAS* (4%, *n* = 1) (Table 1).

### FISH analysis

**Fig. 3:**
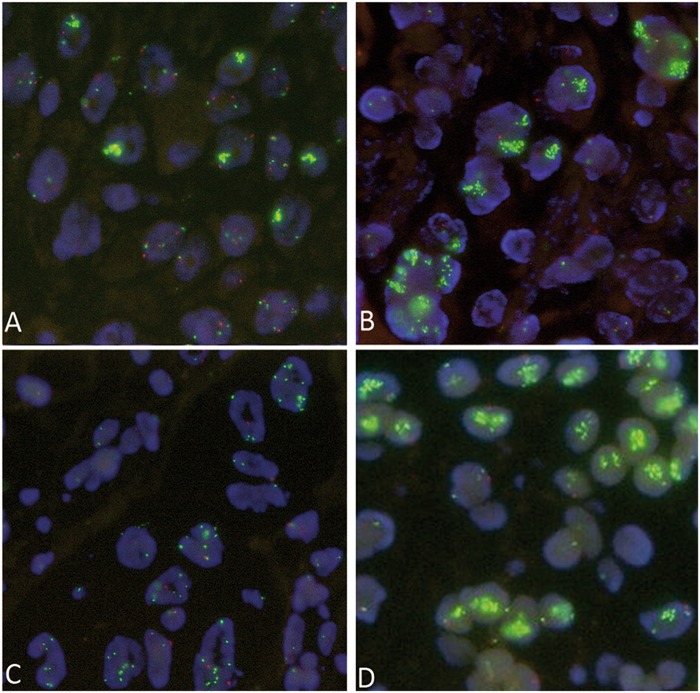
FISH analysis. **a** High-level amplified case showing high intratumoral heterogeneity of *MET* in the FISH analysis. Tumor cell nuclei with big clusters of the *MET* gene alternating with tumor cell nuclei with regular gene copy numbers of *MET*. **b** *MDM2* amplification. **c** *MYC* amplification. **d** *CDK4* amplification. Pictures taken at 63x magnification power

FISH analysis for *ALK*, *ROS1*, and *RET* translocation, as well as *MET* amplification was performed at the time of first diagnosis. Neither *ALK*, *ROS1*, nor *RET* rearrangements were detected in any of the cases in both cohorts. Average *MET* copy number per tumor cell in the *MET*-amplified cohort was between 10 and 21, the ratio *MET*/CEP12 between 3.47 and 9.74. All *MET* FISH results were then reviewed again for this study by two pathologists and the amplification status was confirmed. None of the *MET*-mutant cases showed a co-occurring *MET* amplification (*MET/CEP17* ratio between 0.66 and 1.68, Supplementary Table S3). Interestingly, a high number of *MET* high-level amplified cases (*n* = 12) showed a subclonal *MET* amplification with high heterogeneity among the tumor cells (Fig. 3A). In three metastatic *MET* high-level amplified cases, we were able to perform a FISH analysis on the material of the precedent lung resection. In none of the primary resections, we were able to re-identify the high-level amplification detected in the metastasis.

FISH for *MET* was also performed on an additional 25 therapy-naive lung resection specimens. In none of these cases, a high-level amplification was detected, suggesting that *MET* amplification may be a late event in the process of carcinogenesis.

FISH analysis for *MDM2*, *CDK4*, and *MYC* was performed as validation for Nanostring nCounter copy number alteration analysis (Fig. 3B–D).

### Copy-number variation analysis in the MET-mutant cohort

As shown in Table 1, in the *MET*-mutant cohort we demonstrated an average *MET* gene copy number per cell of two. Recurrent *MDM2* amplifications (27%, *n* = 7) were detected with an average gene copy number per cell of five, with peaks up to 20 gene copies per cell detected in case M5. *CDK4* (23%, *n* = 6), *HMGA2* (19%, *n* = 5), and *DYRK2* (19%, *n* = 5) were also found to be amplified in this cohort. Amplifications of *CDK4* and *MDM2* were confirmed using FISH analysis. Copy number gains were detected for *CCND1* (35%, *n* = 9), *MYC* (15%, *n* = 4), and *TERT* (15%, *n* = 4).

### Copy-number variation analysis in the MET-amplified cohort

The results of copy-number alteration analysis are depicted in Fig. 2 and Supplementary Fig. S1. *MET* high-level amplification detected by FISH showed high concordance with the copy-number alteration analysis results in 16 of 24 cases, in 8 of 24 cases we noted a high discrepancy in the amplification level of *MET*. For all eight discordant cases, we were able to identify either the presence of high intratumoral heterogeneity using FISH analyses or a high number of intratumoral lymphocytes. High-level amplified tumor cells with cluster amplification were identified adjacent to tumor cells with normal signal pattern or lymphocytes with normal signal pattern (Fig. 3A) leading to a lower average gene copy number. When using the Nanostring nCounter^®^ technology, it was not achievable to exclude all normal cells without *MET* alterations. Taken together, both events may explain the discordance between the Nanostring nCounter^®^ technology results and FISH analysis and justify the presence of the *MET* amplification.

*MYC* high-level amplifications (average gene copy number per cell between 5 and 14) were detected in three cases (12%) and *MYC* copy number gains in eight further cases (33%). *CCND1* and *CCND2* showed a recurrent low copy number gain in 14 cases (58%).

As shown in Fig. 2 and Supplementary Fig. S1, both the *MET*-mutant and *MET*-amplified cohort revealed diffuse genetic instability, pointed out on the chart as deviation from average gene copy number of two, but the global amount of copy number alterations was significantly lower in the mutant cohort, when compared with the amplified group (*p* < 0.001), suggesting a reduced genetic instability in the former group.

### Use of MET immunohistochemistry in detecting MET aberrations

Both *MET*-amplified and *MET*-mutant cases were evaluated using immunohistochemistry. The entire cohort of *MET*-amplified cases was evaluated as positive (scores 2–3), confirming the FISH results: six of 24 cases were scored as 2 + and 16 cases as 3 + . In two cases, immunohistochemical evaluation was not possible, due to lack of material.

**Fig. 4:**
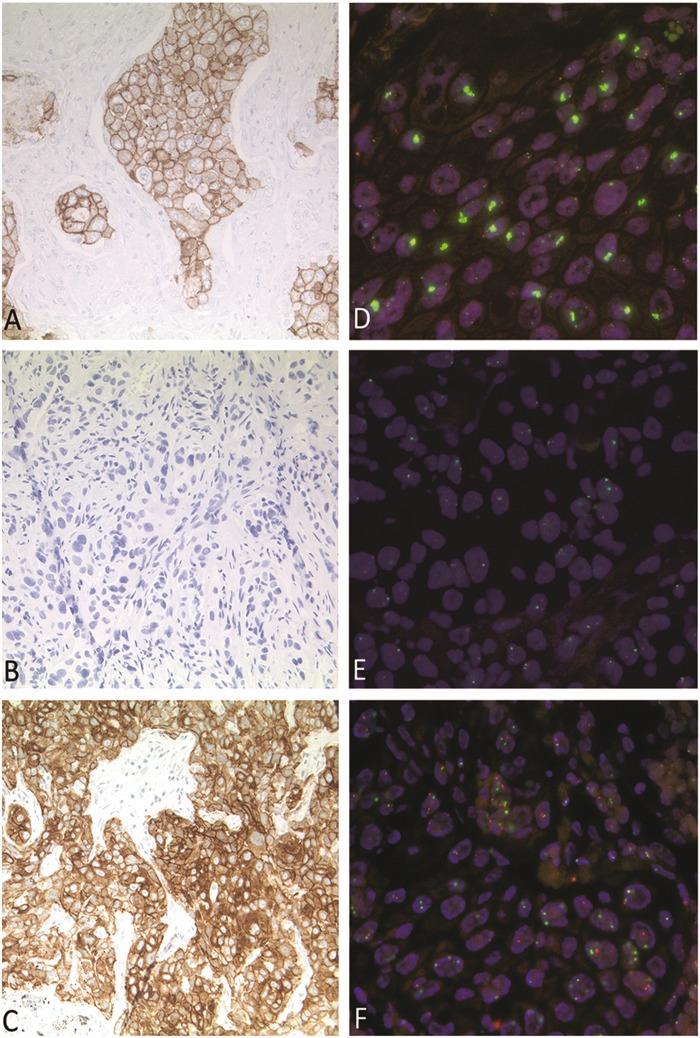
Comparison of MET FISH and immunohistochemistry in *MET*-mutant and -amplified carcinomas. **a**–**c** Immunohistochemical analyses of MET. **a** Example of  *MET* high-level amplified case, score 3+. **b** Example of *MET*-mutant case with negative immunohistochemistry staining, score 1+. **c** Example of *MET*-mutant case with positive immunohistochemistry staining, score 3+. Pictures taken at 20x magnification power. **d**–**f** Paired FISH of *MET*. **d** Ratio *MET*/CEP17 7.36. **e** Ratio *MET*/CEP17 0,66. **f** Ratio *MET*/CEP17 1,18. Pictures taken at 63x magnification power

In contrast, MET immunohistochemistry showed extremely heterogeneous results in the *MET*-mutant cohort (Fig. 4), indicating that immunohistochemistry may not be a suitable prescreening tool in this cohort.

### Survival analysis

**Fig. 5:**
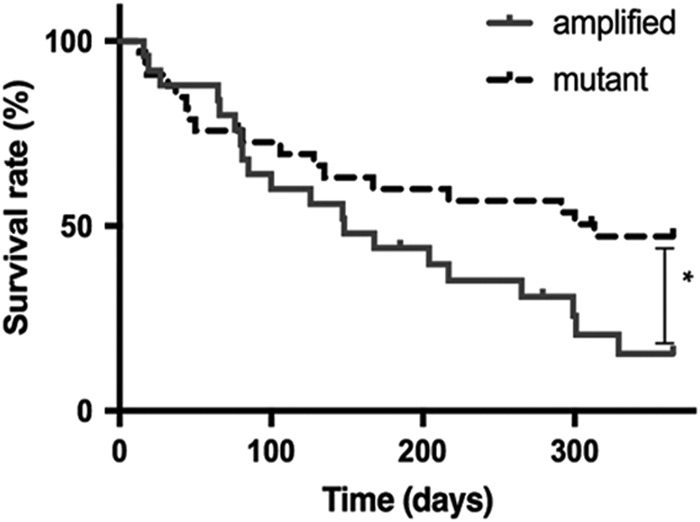
Survival curve at 12 months after diagnosis of stage IIIb/IV for the whole cohort (*n* = 50) of *MET*-altered lung cancer. Amplified: non-small cell lung cancer harboring *MET* high-level amplification. Mutant: non-small cell lung cancer harboring *MET* exon 14 skipping mutations

Stratification of patients by genetic alterations was statistically significant: the subset of *MET*-mutant non-small-cell lung cancer showed a better survival rate at 1 year after diagnosis of disease in advanced stage (stage IIIb/IV) compared with *MET* high-level amplified group (HR = 2.215, 95% CI 1.035–4.74, Fig. 5). The survival analysis showed that patients with a *MET*-mutant carcinoma had an average survival of 456 days, which was significantly longer than the matched group of patients with *MET*-high-level-amplified carcinomas (251 days, *p* = 0.027).

## Discussion

Non-small-cell lung cancer driven by MET aberrations is currently considered as a heterogeneous group of tumors [36]. At the time of this study, no drug targeting MET had been approved by the US Food and Drug Administration, but several ongoing clinical trials are investigating the different MET-activating alterations, using the same inhibitors as INCB028060 [27]. In this context, few studies have investigated side-by-side the genomic background of patients harboring different kinds of MET aberrations. In our study, we genomically and clinically compare two cohorts of non-small-cell lung cancer patients, harboring either *MET* exon 14 skipping mutations or *MET* high-level amplifications, defined by a gene copy number of at least 10.

The analysis of the genomic alterations of lung cancer detected by targeted next-generation sequencing reveals that recurrent *TP53* mutations are present in both groups, however at higher frequency in the cohort of *MET*-amplified cases (*p* = 0,0048). As described in the literature, the higher frequency of *TP53* mutation may be explained by the high prevalence of smokers in this group [43]. Of interest, the *MET*-mutant cohort does not have any co-occurring driver mutations, confirming that *MET* exon 14 skipping mutations may represent a strong carcinogenic driver. Conversely, *MET*-amplified cases frequently show co-occurring driver mutations, such as *KRAS* and *NRAS*, which has also been described previously [29]. Leiser et al. [44] have demonstrated in a preclinical model that *KRAS* mutations in *MET*-amplified cells lead to resistance against MET inhibitors, in a similar way to EGFR-inhibitors. They suggest that the impact of *KRAS* mutations should not be undervalued during treatment planning and could be responsible for reduced/no response after treatment with MET inhibitors. Similar to *KRAS* alterations, we detect the presence of co-occurring high-level *MYC* amplifications in 12 *MET*-amplified cases. *MYC* high-level amplification is also detectable in three *MET*-mutant cases. According to Shen et al. [45], *MYC* amplifications can be responsible for acquired resistance to MET inhibition in MET-addicted cancers. In mouse models, the resistance could be overcome by double-MYC-MET inhibition. Further studies are necessary to clarify the role of *KRAS* mutations and *MYC* amplifications in *MET*-altered non-small-cell lung cancer.

We further analyzed the *MET* signal distribution in the *MET*-amplified cases, using FISH. Interestingly, we show that intratumoral heterogeneity is present at high frequency and is represented by different tumor clones either intermingled or located closely to each other. Of note, it results in a lower level of total gene copy numbers, when using extraction-based copy number variation analysis, compared with single-cell methods, such as FISH (Fig. 3A and Supplementary Fig. S1). Intratumoral heterogeneity may affect clinical outcome, being responsible for reduced treatment response and finally worse clinical outcome. For this reason, FISH remains the gold standard for the evaluation of amplifications in the routine clinical setting.

Furthermore, we describe that the *MET*-mutant group frequently shows co-occurring *CDK4, MDM2, DYRK2*, and *HMGA2* amplifications. These genes co-localize on chromosome 12q, suggesting the presence of an amplification of the entire chromosomal region, rather than co-occurring single gene amplifications.

Using copy-number alteration analysis, we show that high copy-number variation is a more frequent event in *MET*-amplified group, indicating that genomic instability at the chromosomal level is more marked in amplified cases, according to earlier data published by Ciriello et al. [46].

To analyze whether *MET* high-level amplification may represent a late event in the carcinogenesis, we have performed FISH for *MET* on 25 independent cases of therapy-naive resection specimens of non-small-cell lung cancer in early stage: in none of them a high-level amplification is detectable. Furthermore, we have performed FISH for *MET* on two primary non-small-cell lung cancer resection samples showing a *MET* high-level amplification in the metachronous metastasis. No evidence of a high-level amplification is detected in the thoroughly analyzed paired lung resection sample, supporting the idea that *MET* amplification is a late genetic event.

As immunohistochemistry can serve as a reliable screening tool to identify patients with specific genetic alterations, we have also analyzed the MET protein expression level using immunohistochemistry in both groups. We describe positive staining results (score ≥ 2 + ) in all amplified cases. However, MET-immunohistochemistry results are extremely heterogeneous in the mutant cohort, with scores between 0 and 3+. In conclusion, immunohistochemistry seems to be a highly sensitive method for the detection of suspected high-level amplifications. In contrast, it does not seem to be a reliable screening tool for the detection of *MET* exon 14 skipping mutations (Fig. 4).

Finally, the analysis of the clinical features of the cohorts highlights that the two groups differ not only genetically but also clinically. Patients harboring *MET* high-level amplifications are predominantly younger (average age 66 years old, Table 1), male, smokers, compared with patients with *MET* exon 14 skipping mutations, who are in the most cases elderly female, never-smokers (average age 76 years old, Table 1). Described differences in age, sex, and smoking-association are all statistically significant at *p* < 0.05 (Table 1), as also previously described in the literature [26]. The substantial difference in clinical behavior between the two genetic alterations of *MET* is highlighted also by a significantly better survival rate at 1 year after diagnosis for the *MET*-mutant patients (*p* = 0.040) when compared with the amplified patients and by a longer average survival time (*p* = 0.003) as depicted in Fig. 5.

Currently, the therapeutic options for patients affected by *MET*-altered lung cancer in an advanced stage are limited to clinical trials, using crizotinib or other more recently developed MET inhibitors. Our results confirm that *MET*-altered tumors are a biologically heterogeneous group of tumors and we suggest that an accurate characterization of the genetic background may be useful for a better understanding of patients’ outcome with the ultimate goal to improve tailored therapeutic options.

The current study is limited as it is based on the analysis of a restricted number of genes frequently altered in lung cancer and a relatively small number of patients.

Many studies have already described the composition of patient cohorts in *MET*-mutant and -amplified lung cancers, but only few studies directly compare both groups. In spite of sampling number limitations, the study underlines interesting issues that are crucial in both predicting and understanding response to MET inhibitors. This new side-by-side genetic characterization shows that *MET* exon 14 skipping mutations can be interpreted as early strong driver to carcinogenesis in lung cancer cases, whereas *MET* amplifications seem to occur as subclonal genetic event usually in the context of other strong driver mutations and therefore must be interpreted in the context of each tumorʼs genetic background, rather than as isolated event.

The present study provides the scientific basis for the performance of furthermore comprehensive studies analyzing a larger series of cases.

## Electronic supplementary material

Supplementary Table S1

Supplementary Table S2

Supplementary Table S3

Supplementary Figure S1

Supplementary Figure Legend
