# Supplementary material for: Comparison of the genomic background of MET-altered carcinomas of the lung: biological differences and analogies
Source: Mod Pathol. 2018 Nov 20;32(5):627–38. doi: 10.1038/s41379-018-0182-8 (PMC6760650; doi:10.1038/s41379-018-0182-8)
Supplement: Supplementary file 1 — Supplementary Table S1 [file 41379_2018_182_MOESM1_ESM.docx]

| **Antibody** | **Clone** | **Type** | **Dilution** | **Company** | **automated slide-processing platform** |
| --- | --- | --- | --- | --- | --- |
| ALK | clone 1A4 | mouse, monoclonal | 1:100 | Zytomed, Bremerhaven, Germany | Leica BOND-MAX, Leica Biosystems, Wetzlar, Germany |
| PD-L1 | clone28-8, | rabbit, monoclonal | 1:100 | Abcam, Cambridge, UK | Leica BOND-MAX, Leica Biosystems, Wetzlar, Germany |
| MET | clone SP44 | rabbit, monoclonal | ready to use | Ventana, Oro Valley, USA | Benchmark ULTRA, Ventana, Oro Valley, USA |

**Supplementary Table S1.** List of antibodies, clones and dilution used for Immunohistochemical analyses.
