# Supplementary material for: Comparison of the genomic background of MET-altered carcinomas of the lung: biological differences and analogies
Source: Mod Pathol. 2018 Nov 20;32(5):627–38. doi: 10.1038/s41379-018-0182-8 (PMC6760650; doi:10.1038/s41379-018-0182-8)
Supplement: Supplementary file 2 — Supplementary Table S2 [file 41379_2018_182_MOESM2_ESM.docx]

| **Gene inquired** | **Tissue** | **Probe** | **Incubation** | **Company** | **Diagnostic criteria** |
| --- | --- | --- | --- | --- | --- |
| *MET* | 1.5 μm FFPE slide | ZytoLight SPEC MET/CEN7 Dual Color Probe | overnight | ZytoVision GmbH, Bremerhaven, Germany | average GCN ≥10 |
| *ALK* | 1.5 μm FFPE slide | ZytoLight® SPEC ALK/EML4 TriCheckTM Probe | overnight | ZytoVision GmbH, Bremerhaven, Germany | at least 15% of tumor cells show aberrant signals defined as break-apart or extra green |
| *ROS* | 1.5 μm FFPE slide | ZytoLight® SPEC ROS1 Dual Color Break Apart Probe | overnight | ZytoVision GmbH, Bremerhaven, Germany | at least 20% of tumor cells show aberrant signal defined as break-apart or extra green |
| *RET* | 1.5 μm FFPE slide | ZytoLight® SPEC RET Dual Color Break Apart Probe | overnight | ZytoVision GmbH, Bremerhaven, Germany | at least 20% of tumor cells show aberrant signal defined as break-apart or extra green |
| *MDM2* | 1.5 μm FFPE slide | ZytoLight® SPEC MDM2/CEN12 Dual Color Probe | overnight | ZytoVision GmbH, Bremerhaven, Germany | presence of clusters of gene signals |
| *CDK4* | 1.5 μm FFPE slide | ZytoLight® SPEC CDK4/CEN12 Dual Color Probe | overnight | ZytoVision GmbH, Bremerhaven, Germany | presence of cluster of gene signals |
| *MYC* | 1.5 μm FFPE slide | ZytoLight® SPEC MYC/CEN8 Dual Color Probe | overnight | ZytoVision GmbH, Bremerhaven, Germany | presence of cluster of gene signals |

**Supplementary Table S2.** List of probes used for FISH analyses and targeted genes. FFPE: formalin-fixed paraffin-embedded tissue; GCN: gene copy number
