# Supplementary material for: Comparison of the genomic background of MET-altered carcinomas of the lung: biological differences and analogies
Source: Mod Pathol. 2018 Nov 20;32(5):627–38. doi: 10.1038/s41379-018-0182-8 (PMC6760650; doi:10.1038/s41379-018-0182-8)
Supplement: Supplementary file 3 — Supplementary Table S3 [file 41379_2018_182_MOESM3_ESM.docx]

| Patient data | | | | | | **(Immuno)-histologic data** | | | | **NGS - Miseq** | **FISH Analysis** | | | **CNV analysis** |
| --- | --- | --- | --- | --- | --- | --- | --- | --- | --- | --- | --- | --- | --- | --- |
| Patient  Nr. | **Age** | **Gender** | **Smoker** | **Stage at initial diagnosis** | **Survival time** | **Histol.** | **Biopsy** | **PD-L1**  **IHC** | **MET**  **IHC** | **Mutations**  **(Allel frequency, %)** | **Ratio MET/CEN7** | **average MET GCN pro cell** | **Further**  **FISH** | **CNV>4** |
| A1 | 41 | M | 1 | IV | 1202 | A | W | n.a | 3 | *TP53* c.722C>T p.S241F (16.34) | 7.16 | 10.50 | ALK- ROS- RET- | BBC3=5 CDKN1A=5 EEF1A2=6 ERBB2=7 ITGB4=6 MET=5 NKX2-8=6 TP73=6 VEGFA=5 |
| A2 | 77 | M | 1 | IV | 19 | L | P | 5 | 3 | *TP53* c.772G>A p.E258K (23.46) | 4.76 | 12.78 | ALK- ROS- RET- | MET=12 MYC=9 |
| A3 | 76 | M | 0 | IIIB | 676 | A | N | n.a. | 3 | *TP53* c.514G>T p.V172F (10.62) | 7.07 | 13.43 | ALK- ROS- RET- | MET=8 |
| A4 | 70 | M | 0 | IV | 126 | A | W | n.a. | 2 | - | 8.46 | 21 | ALK- ROS- RET- | MET=11 |
| A5 | 68 | M | 1 | IV | 148 | A | T | 2 | 2 | *KEAP1* c.706G>T p.D236Y (55.47)  *TP53* c.463A>C p.T155P (54.09) | 3.86 | 10.10 | ALK- ROS- RET- | C8orf4=5 FGFR1=9 ITGB4=5 MET=11 WHSC1L1=7 |
| A6 | 61 | M | 1 | IB | 204 | A | W | 0 | 3 | *KEAP1* c.1858_1860del p.Q620del (25.03)  *TP53* c.467G>C p.R156P (43.9) | 7.36 | 17.30 | ALK- ROS- RET- | MET=14 |
| A7 | 69 | M | 1 | IV | 76 | A | P | n.a. | 3 | - | 7.71 | 16.32 | ALK- ROS- RET- | MET=11 |
| A8 | 65 | M | 1 | IV | 85 | A | B | n.a. | 3 | - | 4.86 | 16.82 | ALK- ROS- RET- | MET=8 MYC=9 |
| A9 | 50 | M | 1 | IV | 80 | A | T | n.a. | 3 | *PTEN* c.235G>C p.A79P (49.64) | 3.92 | 10.12 | ALK- ROS- RET- | MET=11 MYC=14 |
| A10 | 52 | M | 1 | IV | 147 | A | T | n.a. | 2 | *TP53* c.574C>T p.Q192* (31.95) | 4,16 | 10.55 | ALK- ROS- RET- HER2- | MET=5 |
| A11 | 57 | M | 1 | IV | 168 | A | T | 0 | 3 | *KEAP1* c.[1241A>T; 1243C>T] p.[N414I;R415C] (7.94)  *TP53* c.473G>T p.R158L (7.23) | 8.15 | 15.37 | ALK- ROS- RET- | MET=5 |
| A12 | 71 | M | 1 | IV | 778 | A | T | 0 | 3 | *KEAP1* c.1773G>C p.W591C (22.42)  *TP53* c.742C>T p.R248W (32.72) | 6.35 | 13.23 | ALK- ROS- RET- | MET=8 |
| A13 | 59 | F | 1 | IB | 185 | A | T | n.a. | 3 | *KRAS* c.35G>T p.G12V (13.17)  *TP53* c.643A>G p.S215G (19.26) | 5.33 | 10.40 | ALK- ROS- RET- | MET=3 |
| A14 | 69 | M | 1 | IB | 476 | A | W | 0 | 2 | *KRAS* c.34G>T p.G12C (70.91)  *TP53* c.404G>T p.C135F (87.06) | 7.73 | 13.27 | ALK- ROS- RET- | MET=10 |
| A15 | 70 | M | 1 | IV | 66 | A | T | 4 | 3 | *DDR2* c.404G>A p.R135H (9.18)  *NFE2L2* c.238A>C p.T80P (11)  *TP53* c.464C>T p.T155I (21.58) | 4.78 | 10.20 | ALK- ROS- RET- | MET=4 |
| A16 | 71 | F | 1 | IV | 299 | S | n.a. | 3 | n.a. | *TP53* c.818G>T p.R273L (51.27) | 5.01 | 13.28 | ALK- ROS- RET- | CCND1=5 CCND2=6  MET=22 |
| A17 | 58 | F | 1 | IV | 65 | A | N | 5 | 3 | *NFE2L2* c.241G>T p.G81C (36.68) | 5.12 | 11.01 | ALK- ROS- RET- | CCND1=6 MET=12 MYC=5 NFX2-8=7 PAX9=5 |
| A18 | 72 | F | 1 | IV | 279 | S | N | 0 | 3 | *MET* c.3064_3082+8del (46.15) | 4.49 | 12.13 | ALK- ROS- RET- | DYRK2=9 MAPK7=5  MET=7 NKX2-8=5 |
| A19 | 85 | M | 1 | IV | 81 | A | P | 1 | 3 | *KEAP1* c.1289G>T p.G430V (47.69) | 4.13 | 11.90 | ALK- ROS- RET- | MET=12 |
| A20 | 72 | F | 1 | IV | 329 | A | T | 2 | 3 | *KEP1* c.1258delG p.V420Sfs*38 (33.07)  *TP53* c.569C>T p.P190L (32.02) | 9.74 | 19.73 | ALK- ROS- RET- | CCND1=6 MET=15 NKX2-8=5 |
| A21 | 62 | M | 1 | IA | 88 | A | T | 3 | 2 | *KRAS* c.37G>T p.G13C (43.58)  *TP53* c.800G>T p.R267L (16.72) | 3.47 | 12.50 | ALK- ROS- RET- | MET=10 |
| A22 | 63 | M | n.a. | IVA | 159 | A | T | 5 | 3 | *NRAS* c.173C>T p.T58I (33.98)  *TP53* c.841G>T p.D281Y (51.03) | 4.97 | 11.93 | ALK- ROS- RET- | CDKN1A=18 MET=2 |
| A23 | 86 | M | 1 | IVB | 27 | A | T | 5 | 3 | *KRAS* c.34G>T p.G12C (18.50)  *NFE2L2* c.85G>C p.D29H (11.80)  *TP53* c.722C>T p.S241F (11.86) | 5.25 | 13.22 | ALK- ROS- RET- | CCND1=6 MET=4 NKX2-8=6 SHH=5 |
| A24 | 63 | M | 1 | IV | 265 | A | T | 2 | 3 | *TP53* c.581T>G p.L194R (24.36) | 4.75 | 11.32 | ALK- ROS- RET- | CCND1=6 MET=5 NKX2-8=5 |
|  |  |  |  |  |  |  |  |  |  |  |  |  |  |  |
| M1 | 72 | M | n.a. | IB | 724 | A | W | n.a. | 1 | *MET* c.3082+1G>T (13.88) | 0.98 | 2.12 | ALK- ROS- RET- | MDM2=6 |
| M2 | 67 | F | 0 | IV | 502 | A | P | 5 | 1 | *EGFR* c.2314C>T p.P772S (2.66)  *MET* c.3082+1G>T (18.96) | 0.98 | 1.35 | ALK- ROS- RET- | - |
| M3 | 77 | F | n.a. | IV | 44 | A | T | 2 | 3 | *MET* c.3082+1G>C (28.9)  *TP53* c.733_737delins5bp p. [G245C(;)M246T] (50.37) | 1.41 | 1.85 | ALK- ROS- RET- | - |
| M4 | 77 | F | n.a. | IV | 313 | A | T | n.a. | 0 | *MET* c.2942-24_2942-13del (23.62) | 1.00 | 2.45 | ALK- ROS- RET- | - |
| M5 | 68 | F | 0 | IV | 106 | A | N | 3 | 0 | *MET* c.3082+2T>C (12.81) | 0.66 | 1.63 | ALK- ROS- RET- | CDK4=7 DYRK2=13 HMGA2=5 MDM2=20 |
| M6 | 79 | F | 1 | IV | 923 | A | T | 5 | n.a. | *MET* c.2942-16_2944del (20.41) | 1.68 | 2.85 | ALK- ROS- RET- HER2- | - |
| M7 | 77 | M | 0 | IIIB | 753 | A | T | 1 | 3 | *MET* c.3082+1G>T (25.38) | 1.39 | 3.62 | ALK- ROS- RET- | - |
| M8 | 75 | F | 0 | IV | 724 | A | T | 0 | 1 | *MET* c.2942-19_2942-3del (6.06) | 1.01 | 2.47 | ALK- ROS- RET- | CDK4=7 HMGA2=7 MDM2=10 |
| M9 | 68 | F | 1 | IV | 658 | A | T | 5 | 1 | *MET* c.3082G>C p.D1028H  (12.24) | 0.89 | 2.10 | ALK- ROS- RET- | - |
| M10 | 87 | M | 0 | IV | 541 | A | T | 1 | 2 | *MET* c.3082+2T>C (15.37)  *TP53* c.617delT p.L206Wfs*41 (9.73) | 0.89 | 2.22 | ALK- ROS- RET- | - |
| M11 | 85 | M | 0 | IA | 505 | A | T | 3 | 3 | *MET* c.3082+2T>C (11.27) | 1.36 | 3.63 | ALK- ROS- RET- | HMGA2=8 MDM2=7 SKP2=6 TERT=10 |
| M12 | 82 | M | 0 | IV | 81 | A | T | 5 | 3 | *MET* c.3082+2T>C (28.02) | 1.31 | 3.83 | ALK- ROS- RET- | MYC=5 |
| M13 | 88 | F | n.a. | IV | 1781 | A | W | 0 | 2 | *MET* c.3082+1G>T (6.83) | 1.18 | 4.35 | ALK- ROS- RET- | - |
| M14 | 57 | F | 0 | IV | 45 | A | T | 5 | 3 | *MET* c.3082+1G>T (20.99) | 1.27 | 2.60 | ALK- ROS- RET- | - |
| M15 | 83 | F | 0 | IVB | 50 | A | T | 5 | n.a. | *MET* c.3082G>C p.D1028H (6.56)  *TP53* c.853G>A p.E285K (8.51) | 1.14 | 2.82 | ALK- ROS- RET- | CCND1=6 NKX2-8=6 |
| M16 | 64 | M | 1 | IVB | 418 | A | T | 5 | 3 | *MET* c.3082+1G>T (6.83) | 1.15 | 3.07 | ALK- ROS- RET- | - |
| M17 | 79 | M | 1 | IV | 78 | A | T | 0 | 0 | *MET* c.3082_3082+23del (30.68) | 1.40 | 3.77 | ALK- ROS- RET- | CDK4=9 DYRK2=8 MDM2=7 MYC=7 |
| M18 | 85 | F | 1 | IV | 531 | A | T | 5 | 0 | *MET* c.2942-14_2963del (14.84)  *TP53* c.469G>T p.V157F (17.24) | 1.54 | 3.08 | ALK- ROS- RET- | - |
| M19 | 74 | M | 1 | IV | 517 | A | W | 0 | 0 | *MET* c.2942-2_2942-20del (10.5)  *TP53* c.511G>A p.E171K (8.63) | 1.25 | 2.72 | ALK- ROS- RET- | - |
| M20 | 85 | M | 0 | IV | 171 | A | T | 5 | 0 | *MET* c.2942-18_2942-4del (3.69) | 0.86 | 1.92 | ALK- ROS- RET- | - |
| M21 | 79 | M | 1 | IIIB | 300 | S | N | 3 | 3 | *MET* c.3082+3A>G (6.07) | n.a. | n.a. | ALK- ROS- RET- | DYRK2=7 MDM2=5 |
| M22 | 69 | M | 1 | IV | 50 | A | T | 0 | 3 | *MET* c.2942-7_2942-17delinsC (8.2) | 1.61 | 2.98 | ALK- ROS- RET- | - |
| M23 | 72 | F | 0 | IIIB | 128 | A | T | 5 | 2 | *MET* c.3082+3A>G (6.38)  *TP53* c.447delC p.T150Hfs*20 (16.94) | 1.22 | 3.52 | ALK- ROS- RET- | CRKL=6 |
| M24 | 73 | M | 0 | IV | 311 | A | W | 0 | 3 | *MET* c.2942-28_2942-2del (4.6) | 1.02 | 2.13 | ALK- ROS- RET- | DYRK2=5 HMGA2=5 MDM2=12 |
| M25 | 81 | F | 0 | IV | 135 | S | T | 3 | 1 | *MET* c.3082+3A>G (6.91) | n.a. | n.a. | ALK- ROS- RET- | - |
| M26 | 67 | F | n.a. | IV | 1474 | A | n.a. | n.a. | n.a. | *MET* c.2942-19_2942-9del (38.1) | 1.01 | 2.40 | ALK- ROS- RET- | TERT=6 |

**Supplementary Table S3. Histological and molecular characteristics.** Patient Nr. A: amplified, M: mutated. Age. Age expressed in years at time of stage IIIb/IV diagnosis. Gender. F: female, M: male. Smoker. 0: no, 1: yes. Stage at initial diagnosis. Stage at first diagnosis according to Union for International Cancer Control (UICC) guidelines. Survival time. Survival time expressed in days from the time of diagnosis of stage IIIb/IV. Histology. A: adenocarcinoma, L: large cell carcinoma, S: squamous. Biopsy. B: bone piopsy, N: needle core biopsy, P: pleural biopsy, T: transbronchial biopsy, W: wedge biopsy. PD-L1-immunohistochemistry (IHC). PD-L1 scored according to „Cologne scoring system“, score 0: <1% positive tumor cells; 1: ≥1%, <5%; 2: ≥5%, <10%; 3: ≥10%, <25%; 4 ≥25%, <50%; 5 ≥50%. MET-IHC. Score 3: ≥ 50% of tumor cells stained with strong intensity; score 2:  ≥ 50% of tumor cells with moderate or higher staining but < 50% with strong intensity; score 1: ≥ 50% of tumor cells with weak staining but < 50% with moderate or higher intensity; score 0: no staining or < 50% of tumor cells with any intensity. Ratio MET/centromere of chromosome 7(CEP7): cut-off 2.0. MET gene copy number (GCN): cut-off 10.0. Next-generation sequencing (NGS) Miseq. “-“: all genes wt. Further FISH. “-“: negative, “+”: positive. Copy number variation (CNV) analysis. ”-“: all genes showed gene copy number (GCN) ≤4.
