# Supplementary figures and images for: Comparison of the genomic background of MET-altered carcinomas of the lung: biological differences and analogies
Source: Mod Pathol. 2018 Nov 20;32(5):627–38. doi: 10.1038/s41379-018-0182-8 (PMC6760650; doi:10.1038/s41379-018-0182-8)

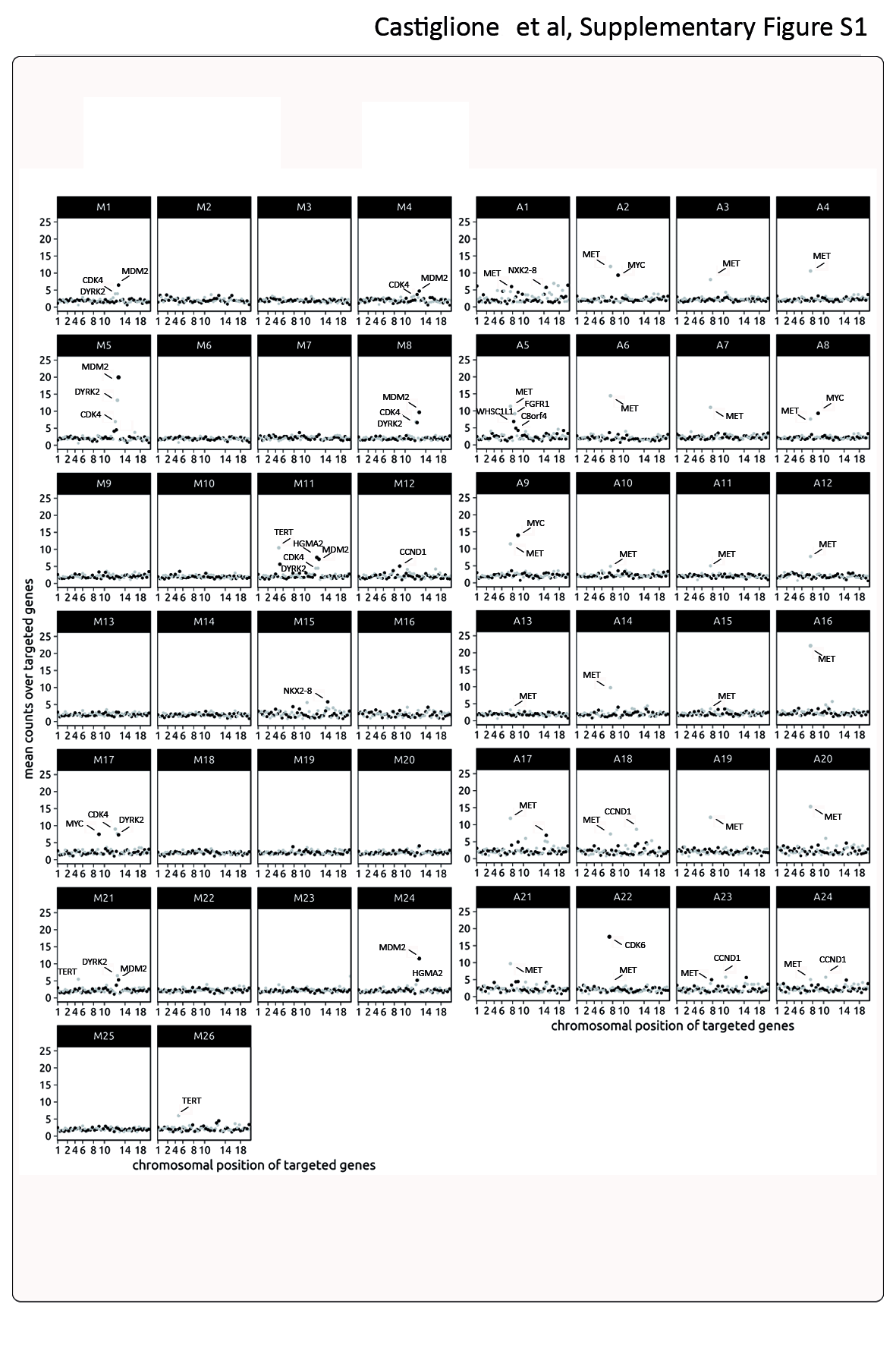

Supplement: Supplementary file 4 — Supplementary Figure S1 [file 41379_2018_182_MOESM4_ESM.tif]
