# Supplementary material for: Comparison of the genomic background of MET-altered carcinomas of the lung: biological differences and analogies
Source: Mod Pathol. 2018 Nov 20;32(5):627–38. doi: 10.1038/s41379-018-0182-8 (PMC6760650; doi:10.1038/s41379-018-0182-8)
Supplement: Supplementary file 5 — Supplementary Figure Legend [file 41379_2018_182_MOESM5_ESM.docx]

Legend Supplementary Figure S1

**Supplementary Figure S1**. Copy number variation analysis of the cohort of *MET*-altered non-small cell lung cancer. The x-axis indicates the chromosomal position of the genes analyzed, the y-axis the mean gene copy number. Each point represents a single gene. Cases M: MET-mutant; cases A: MET high-level amplified.
